# Psychopathological Correlates of Dysfunctional Smartphone and Social Media Use: The Role of Personality Disorders in Technological Addiction and Digital Life Balance

**DOI:** 10.3390/ejihpe15070136

**Published:** 2025-07-17

**Authors:** Mirko Duradoni, Giulia Colombini, Camilla Barucci, Veronica Zagaglia, Andrea Guazzini

**Affiliations:** Department of Education, Literatures, Intercultural Studies, Languages and Psychology, University of Florence, 50135 Florence, Italy; giulia.colombini@unifi.it (G.C.); andrea.guazzini@unifi.it (A.G.)

**Keywords:** personality disorders, internet addiction, digital life balance, social media, borderline personality, narcissistic traits

## Abstract

Current technological development has made the Internet and new technologies increasingly present in people’s lives, expanding their opportunities but also potentially posing risks for dysfunctional use. This study aims to identify psychopathological factors associated with dysfunctional ICT use, extending the evidence beyond the well-established relationships with mood disorders to include personality disorders (i.e., cluster C in particular). A total of 711 participants (75.70% female; Mage = 28.33 years, SD = 12.30) took part in the data collection. Firstly, the results showed positive correlations between higher levels of addictive patterns for the Internet, social networks, smartphones and applications, and video games and higher levels of borderline symptoms as assessed by the Borderline Symptom List 23—Short Version. Moreover, scores reflecting high addictive patterns also positively correlated with general narcissistic traits as indicated by the total score of the Narcissistic Personality Inventory 13—Short Version and those specifically described by its Entitlement/Exploitativeness dimension, as well as with higher levels of almost all the personality traits assessed by the Personality Inventory for DSM 5—Brief Form (i.e., negative affectivity, detachment, disinhibition, and psychoticism). These findings broaden the still scarce body of evidence on the relationship between personality disorders and dysfunctional ICT use, which, however, needs to be further explored.

## 1. Introduction

The technological revolution, driven by mobile technology and the Internet, has opened up widespread opportunities. Greater access to digital tools has set in motion a complex transformation of the self and human relationships (Brubaker, 2020; Hynes, 2024), enabling people to use services that were once only available offline (e.g., learning, telemedicine, home banking) and to experience life moments, satisfy needs, and engage in personal and social processes online (Duradoni et al., 2024a). In this context, however, the risk of dysfunctional patterns of use may emerge; thus, psychological research on the use of new technologies is increasingly focused on understanding these particular dynamics. For example, some scholars have investigated the mediating role of flow and media multitasking in the development of problematic smartphone use (Wickord & Quaiser-Pohl, 2025). Due to their technological affordances, smartphones are capable of immersing users in a state of flow, highly rewarding experiences that may encourage excessive use over time (C. Chen et al., 2017). This effect can be further intensified by media multitasking, which allows users to engage in multiple simultaneous activities, each potentially triggering further flow. In these states, people may lose track of time and use devices longer than intended. Gong et al. (2021) suggest that such experiences may act as a coping strategy for stress, increasing the risk of Internet addiction.

Next to this evidence, other studies have explored the link between specific psychological conditions and dysfunctional technology use, focusing in particular on mood disorders. Some findings have identified depression as a potential predictor of mobile addiction (Jamir et al., 2019), which is also positively associated with social media addiction across countries (Dailey et al., 2020; Haand & Shuwang, 2020). Indeed, people with depression or anxiety sometimes report higher levels of social media addiction than control groups despite similar usage frequency (Şentürk et al., 2021). These patterns are commonly explained by efforts to escape negative moods or modify mood (Caplan, 2002). Moreover, people with depression may also prefer online interactions over face-to-face communication to fulfill their social needs, as their condition often reduces their offline social engagement (Caplan, 2002; Satici, 2019; Aydin et al., 2020; Kupferberg et al., 2016; Turel et al., 2011).

In this perspective, recently the Psychology of Harmony and Harmonization framework (Di Fabio & Tsuda, 2018) has posited that unmet real-life needs, which find satisfaction online, may drive dysfunctional technology use, underpinning the concept of Digital Life Balance (DLB), that is, harmonious ICT use (Duradoni et al., 2022). Supporting this, studies have shown that fulfilling psychological needs online, especially social and control ones, can foster dysfunctional usage patterns that harm overall well-being (Duradoni et al., 2024b, 2024c). For instance, lower DLB has been found to be associated with higher FoMO, suggesting that compromised social needs or fear of exclusion may increase the risk of developing addictive online behaviors (Duradoni et al., 2024a). Furthermore, people who primarily satisfy their need for mattering through online interactions have been found to be more vulnerable to social media addiction and to report lower levels of Digital Life Balance (Duradoni et al., 2024c). These findings underscore the importance of psychological differences and needs in shaping how technology is used.

Extending beyond mood-related vulnerabilities to include personal differences as well, emerging research has suggested a link between personality disorders and problematic technology use. While traits from Cluster B and C have been associated with a higher risk of Internet addiction (Zadra et al., 2016), this relationship remains underexplored. From a needs-based perspective, research has primarily focused on narcissistic traits, emphasizing their role in fulfilling social and emotional regulation needs through technology (Billieux et al., 2015; Pantic et al., 2017; Zadra et al., 2016).

In the area of social needs, people with narcissistic traits tend to use social media more frequently and are more concerned with maintaining their popularity (Pantic et al., 2017; Şentürk et al., 2021). These behaviors are likely driven by the need for social approval, admiration, and attention, which are positively associated with social media use (Savci et al., 2021) and smartphone addiction (Pearson & Hussain, 2016; Zerach, 2021). On social platforms, people with narcissistic personality traits also tend to focus on receiving feedback, such as likes and recognition, often performing self-promotional behaviors like frequent selfie-posting (Andreassen et al., 2017). Consistently, recent findings point to the role of a heightened need for online social feedback (Duradoni et al., 2023). These dynamics reflect the drive for admiration in people with narcissistic traits, as social media boosts self-esteem (Pantic et al., 2017) and reinforces these traits through likes and interactions (Cıkrıkcı & Yalcın, 2023; Kim et al., 2008). In the context of online gaming, narcissistic traits have also been linked to the pursuit of power and status, mirroring real-life behaviors in virtual settings and further boosting self-worth (Kim et al., 2008). Moreover, their digital usage may be prompted by a need for connection and reassurance (Billieux et al., 2015), which is often associated with insecure attachment, anxiety (X. Lu et al., 2014), and low self-esteem (Ehrenberg et al., 2008). This is especially evident in people with vulnerable narcissism, who tend to seek excessive reassurance through Internet and smartphone use (Zerach, 2021). Compared to grandiosity, vulnerable narcissism appears to play a key role in problematic smartphone use, likely due to underlying feelings of emptiness (Zerach, 2021). This highlights the need to consider vulnerability when assessing risk for smartphone addiction. Along these lines, Wink and Donahue (1997) found an association between vulnerable narcissism and boredom. Following Ksinan et al. (2021), boredom may mediate the relationship between narcissistic vulnerability and compulsive smartphone use.

Addictive dynamics may also stem from emotional regulation needs (Zadra et al., 2016), reducing negative self-awareness or escaping unresolved painful feelings like abandonment, worthlessness, and emptiness, that people with narcissistic traits may potentially fear (Nixon, 2013).

Similar need-driven mechanisms are evident in people with borderline personality traits, where anxiety often leads to interpersonal reassurance-seeking behaviors through frequent Internet use to bolster self-esteem and reduce worries and uncertainty (Clerkin et al., 2013). According to T.-H. Chen et al. (2019), it is mainly the level of identity impairment that would predict the risk of Internet addiction, although other borderline features such as impulsivity, unstable relationships and feelings of emptiness and loneliness may also contribute to an increased risk (W.-H. Lu et al., 2017). In fact, the Internet is often used as a coping mechanism for emotional distress and negative moods (Caplan, 2002; Dalbudak et al., 2014; Wu et al., 2016). In this context, positive expectations of stress relief may mediate the link between borderline traits and problematic technology use, reflecting an attempt to compensate for poor social skills. Furthermore, impulsivity, a central feature of borderline personality disorder, can expose people with these traits to a higher risk of developing a problematic relationship with technology. Evidence supporting this association has been found in studies on Internet addiction (Cao et al., 2007), social media addiction, and problematic smartphone usage (Guo et al., 2022). These links have been associated with addictive behaviors involving mood modification, relapse, and withdrawal due to difficulties with emotion regulation and response inhibition (Guo et al., 2022).

Research in this area has also found that the personality traits assessed by the Personality Inventory for DSM 5 (PID-5; Fossati et al., 2024), namely disinhibition, negative affectivity, detachment, psychoticism, and antagonism, may also constitute a vulnerability to the development of Internet addiction (Gervasi et al., 2017; Laier et al., 2018; Müller et al., 2021; Romero & Alonso, 2019; Venuleo et al., 2021). In particular, disinhibition is often marked by impulsivity and antisocial tendencies (Romero & Alonso, 2019). As reported by Romero and Alonso (2019), according to the online disinhibition effect (Suler, 2004), the performance of such behaviors seems to be facilitated by the use of the Internet, especially by anonymity, increasing online engagement. The Internet’s immediacy may ease impulsive tension, supporting its compensatory use as a coping strategy (Gervasi et al., 2017; Kardefelt-Winther, 2014). This dynamic may contribute to other dysfunctional patterns such as nomophobia, short for “No MObile PHone phoBIA” (King et al., 2013). It is defined as the anxiety or discomfort experienced when people are unable to use their electronic devices (Rodríguez-García et al., 2020) or to access virtual communications environments (Yildirim & Correia, 2015). Although the current literature on this phenomenon remains limited as compared to research on personality disorders, one study has indicated a potential association between nomophobia, neuroticism, and disinhibition (Dib et al., 2022). For this reason, it is worth exploring this trait from a need-based perspective, such as that of DLB.

Negative affectivity has also been linked to increased Internet use, particularly among people with social anxiety, as online interactions involve less emotional activation (Venuleo et al., 2021) and provide greater control over self-presentation (Mehdizadeh, 2010; Peter et al., 2007). The absence of immediate, visible feedback (Leigh & Clark, 2018) can help regulate negative affectivity and reduce emotional lability (Liu & Campbell, 2017). Such a compensatory function (Kardefelt-Winther, 2014) could, in the long run, motivate people toward increasingly dysfunctional Internet use (Gervasi et al., 2017; Venuleo et al., 2021).

Similarly, the trait of detachment, which is characterized by social and emotional avoidance behaviors, has been associated with higher Internet use, as online environments facilitate them (Laier et al., 2018). Indeed, avoidance expectancies have been shown to mediate the relationship between this personality trait and Internet addiction risk. The literature has also shown that avoidance of emotional and social situations can be associated with negative emotional activation and frustrated social needs (Müller et al., 2021). This makes the Internet a coping tool (Kardefelt-Winther, 2014) that enables limited interactions in a controllable environment, providing emotionally manageable situations (Müller et al., 2021).

Following the PID-5 dimensions, people with psychoticism traits may also show a tendency toward dysfunctional Internet use (Gervasi et al., 2017), which warrants further analysis as evidence is still sparse. Although there is no evidence in the literature, Internet mechanisms such as the filter bubble (Pariser, 2011) and echo chamber effects (Sunstein, 2001) may help to explain this association. Specifically, the filter bubble exposes users to information that aligns with their beliefs (Pariser, 2011), potentially confirming eccentric ideas that might otherwise be rejected in offline contexts. Similarly, echo chambers increase the likelihood of interacting with like-minded people (Avin et al., 2024), which can fulfill the social identity needs of people higher in psychoticism that might otherwise go unmet in real life (Reinhard et al., 2022).

Finally, the relationship between antagonistic traits and dysfunctional Internet use can be explained by the Theory of the Online Disinhibition Effect (Suler, 2004), for which the disinhibiting effects of online anonymity can facilitate aggressive antisocial behavior (Romero & Alonso, 2019; Yang, 2012). People high in antagonism are characterized by low levels of agreeableness, which contributes to decreasing the quality of their social relationships (Tov et al., 2016). As a result, the Internet may compensate for unmet social needs, such as belonging and affiliation, which are lacking in offline interactions (Tov et al., 2016).

Overall, the existing body of research underscores the necessity of further exploring the relationship between psychopathological personality traits and dysfunctional technology use. These behaviors may serve as maladaptive strategies to satisfy unmet psychological needs that should be acknowledged in order to support individual well-being, as outlined in the Digital Life Balance framework. However, the current literature largely lacks a need-based conceptualization of these dynamics, which limits our understanding of how individual differences may influence digital behaviors and their impact on mental health. On this basis, we developed the aim and the hypothesis that guided our study.

### Aim of the Study and Hypotheses Development

Since the literature primarily highlights associations with mood disorders, this research aims to broaden the perspective by specifically identifying personality psychopathological factors associated with dysfunctional ICT use (i.e., negative affectivity, detachment, antagonism, disinhibition, psychoticism, borderline personality symptoms, and narcissistic personality traits). In fact, Cluster B and Cluster C personality traits and disorders have also been shown to increase susceptibility to certain technological addictions (Zadra et al., 2016).

Recent findings suggest that people with Internet addiction often have higher rates of personality disorders, indicating that psychopathological symptoms and socially maladaptive behavioral traits may increase vulnerability to Internet addiction (Zadra et al., 2016). In light of this and drawing on the Psychology of Harmony and Harmonization (Di Fabio & Tsuda, 2018) and the theory of compensatory Internet use (Kardefelt-Winther, 2014), in our study we selected narcissistic personality disorder, borderline personality disorder, and the traits of disinhibition, negative affectivity, detachment, psychoticism, and antagonism to examine their patterns of association with addiction measures and DLB.

As people with borderline personality tend to use the Internet as a tool to alleviate frequently experienced negative emotions, to compensate for poor social skills, or as a way to cope with psychological distress through dissociation and escape (Wu et al., 2016), we expect the following:

- H1: people with higher borderline personality symptoms will report lower DLB scores and higher new technology addiction scores.

Given that the Internet allows people with narcissistic personality disorder to reinforce self-esteem (Pantic et al., 2017) and to satisfy their needs for admiration (Cıkrıkcı & Yalcın, 2023; Kim et al., 2008) and affiliation (Billieux et al., 2015), as well as their emotion regulation needs (Zadra et al., 2016), we expect the following:

- H2: people with higher narcissistic scores will report lower DLB scores and higher new technology addiction scores.

Since people with personality traits of disinhibition tend to use the Internet to give vent to their impulsivity (Gervasi et al., 2017) but also to facilitate the enactment of antisocial behavior (Romero & Alonso, 2019), we expect the following:

- H3: people with higher disinhibition scores will report lower DLB scores and higher new technology addiction scores.

Considering that people with negative affectivity personality traits are more likely to use the Internet as a tool to alleviate negative emotional activation (Leigh & Clark, 2018), we expect the following:

- H4: people with high negative affectivity scores will report lower DLB scores and higher new technology addiction scores.

Given that the Internet appears to facilitate social withdrawal and avoidance expectations in people with personality traits of detachment (Laier et al., 2018), we expect the following:

- H5: people with high detachment scores will report lower DLB scores and higher new technology addiction scores.

As that Internet anonymity may promote aggressive antisocial behavior in people with antagonistic personality traits (Romero & Alonso, 2019; Yang, 2012), we expect the following:

- H6: people with high antagonism scores will report lower DLB scores and higher new technology addiction scores.

Moreover, thanks to the filter bubble effect (Pariser, 2011) and the echo chamber effect (Sunstein, 2001), it may be easier for people with psychotic personality traits to find acceptance for their eccentric ideas. Therefore, we expect the following:

- H7: people with higher scores of psychoticism will report lower DLB scores and higher new technology addiction scores.

## 2. Methods

### 2.1. Participants and Procedure

Prior to participant recruitment, we conducted a power analysis to estimate the necessary sample size for testing the study’s hypotheses, using G*Power software 3.1 (Faul et al., 2007, 2009). Given that correlation would be the primary inferential test, we aimed to determine the number of participants required to achieve a statistical power of 0.80. This power level was chosen to reliably detect small effects (r = 0.10) with a significance threshold of 0.05. The results of the power analysis indicated that a sample size of 616 participants would be sufficient to meet these criteria. This study was conducted with a total sample size of 711 participants, employing a non-random snowball sampling technique. Data was collected by using Google modules to create an online questionnaire, which we distributed via email and across various online platforms and social networks, including Instagram, Facebook, WhatsApp, and Telegram. Participation was voluntary, as indicated by specific online recruitment messages, with inclusion criteria of a minimum age of 14 and proficiency in the Italian language. Each participant was informed of their right to withdraw from the study at any point. To ensure confidentiality, all collected data were anonymous in line with the Italian privacy legislation (Legislative Decree DL-101/2018) and the EU General Data Protection Regulation (2016/679). The questionnaire required approximately 20 min to complete. The demographic composition of the sample was predominantly cisgender women (75.70%), with ages ranging from 15 to 80 years (mean age = 28.33; SD = 12.30). Of the 711 respondents, 674 reported using at least one social media platform, and 246 affirmed playing at least one video game (approximately 34.6% of the sample). While this does not necessarily indicate regular gaming behavior, this group can be considered representative of people with some gaming experience.

The generalizability of our findings may be constrained by the sample’s characteristics, as it predominantly consists of young individuals (mean age = 28.33) with a high level of education (52.60% holding a secondary school diploma and 30.40% a bachelor’s degree) and a majority of cisgender females (75.70%).

### 2.2. Measures

To achieve the research objectives, an online survey was created and administered using Google Forms. First, each participant was asked for brief socio-demographic information (i.e., age and gender). Then the following measures were used, taking into account for all of them their validated Italian version:

*Digital Life Balance Scale* (DLB) (Duradoni et al., 2022) was composed of 4 items using a 7-point Likert scale, from strongly disagree (1) to strongly agree (7). Examples of items are as follows: “I currently have a good balance between the time I spend online and the time I have available for offline activities” and “Overall, I believe that my online and offline life are balanced”. The reliability of the DLB scale was measured using McDonald’s Omega (JASP ver. 0.16.4.0) and was found to be excellent (ω = 0.89). Possible scores on the scale range from a minimum of 4 to a maximum of 28, and the higher the score, the better the balance between online and offline life. Specifically, this scale aims to capture individuals’ well-being in terms of the perceived balance between their online and offline lives. Unlike established measures of ICT balance, the DLB scale captures both harmonious (balanced) and disharmonious (imbalanced) states by focusing on the role of unmet offline needs in the process of disharmonization between online and offline lives.

*Internet Addiction Scale* (IAS) (Karadağ et al., 2015). This scale measures the presence and severity of Internet addiction. The scale consists of 6 items (e.g., “I feel anxious when I don’t have Internet access”, “I spend more time on the Internet than planned”) that are rated using a 5-point Likert scale (1 = strongly disagree; 5 = strongly agree). Italian items have been preliminarily validated by Guazzini and colleagues (Guazzini et al., 2019), and all the items load onto only one factor. Cronbach’s alpha coefficient for the Internet addiction scale was 0.83 (Guazzini et al., 2019), and the scoring range varies between a minimum of 6 and a maximum of 30, where high scores indicate greater addiction.

*Bergen Social Media Addiction Scale* (BSMAS) (Andreassen et al., 2016). This scale, translated and validated in Italian by Monacis and colleagues (Monacis et al., 2017), is useful for measuring a person’s degree of dependence on social networks. It is made up of 6 items (e.g., “I don’t get tired of playing video games”, “I lose track of time when I play”) that are rated using a 5-point Likert scale (1 = strongly disagree; 5 = strongly agree). The internal consistency coefficients for the scale range from α = 0.88 (Monacis et al., 2017) to α = 0.86 (Shin, 2022). The scoring range varies between a minimum of 6 and a maximum of 30, where high scores correspond to greater addiction.

*Smartphone Application-Based Addiction Scale* (SABAS) (Csibi et al., 2018). It was produced to study a person’s degree of dependence on smartphones and applications. For our studies, we used the version validated in Italian by Soraci and colleagues (Soraci et al., 2021). The SABAS consists of 6 items (e.g., “My smartphone is the most important thing in my life”, “I feel the need to spend more and more time using my smartphone”) that are rated on a 6-point Likert scale (1 = strongly disagree; 6 = strongly agree). The internal reliability of the scale is good, with Cronbach’s alpha coefficients of 0.81 for the original version (Csibi et al., 2018) and 0.89 for the Italian version (Soraci et al., 2021). The scoring range varies between a minimum of 6 and a maximum of 36, where high scores indicate greater addiction. The potential construct distinctiveness issues between IAS and SABAS, as well as their implications for the results, are discussed further in the limitations section.

*Gaming Addiction Scale* (GAS) (Karadağ et al., 2015). It measures the degree of dependence on video games and consists of 8 items (e.g., “I lose track of time when I play”, “I postpone bedtime to play”) that are rated using a 5-point Likert scale (1 = strongly disagree; 5 = strongly agree). Italian items have been preliminarily validated by Guazzini and colleagues (Guazzini et al., 2019), and all the items load onto only one factor. The Cronbach’s alpha coefficient for the GAS was 0.90 (Guazzini et al., 2019), and the scoring range varies between a minimum of 8 and a maximum of 40, where high scores correspond to greater addiction.

We decided to include a gaming addiction measure in our analysis to further explore need satisfaction dynamics through this specific behavior. Some research has highlighted distinct behavioral patterns associated with personality traits (Kim et al., 2008), making gaming another relevant domain for understanding dysfunctional technology use.

*Narcissistic Personality Inventory 13—Short Version* (NPI-13) (Gentile et al., 2013) for the identification of narcissistic personality traits. It is an abbreviated form of the full version of NPI-40 (Raskin & Hall, 1979). This study used the Italian short form, a more up-to-date version than the original, though not yet officially validated (Longo, 2023), which could represent one of the study’s limitations.

Longo (Longo, 2023), based on the German validation (Brailovskaia et al., 2019), proposes a research design for validating this scale in Italian. The scale consists of 13 items with a binary system of answers (0 = low narcissism; 1 = high narcissism). The scale allows for extracting three subscales in addition to the total scale: Leadership/Authority (LA) (“I like having authority over other people” vs. “I don’t mind following orders”), Grandiose/Exhibitionism (GE) (“My body is nothing special” vs. “I like to look at my body”), and Entitlement/Exploitativeness (EE) (“I find it easy to manipulate people” vs. “I don’t like it when I find myself manipulating people”). Cronbach’s alpha coefficient was 0.66 for the LA subscale, 0.65 for the GE subscale, and 0.51 for the EE dimension. The LA and EE dimensions have a score range from 0 to 4; the GE dimension can range from 0 to 5. The total score can range from 0 to 13, where higher scores indicate more pronounced narcissistic personality traits.

*Borderline Symptom List 23—Short Version* (BSL-23) (Bohus et al., 2008) is a 23-item self-rating instrument for specific assessment of borderline personality disorder symptomatology. This scale is an abbreviated form of the full version of BSL-95 (Bohus et al., 2007). In this study, the Italian short form was used (Manco, 2008). Items (e.g., “I hated myself”, “My mood rapidly cycled in terms of anxiety, anger, and depression”) are rated using a 5-point Likert scale (0 = not at all; 4 = very strong). The internal consistency coefficient for the scale is α = 0.96 (Bohus et al., 2008). The average score of items (range 0 to 4, sum of scores divided by 23) is calculated, with a higher score indicating more impairment.

*The Personality Inventory for DSM 5—Brief Form* (PID-5-BF) is an instrument for assessment of the five pathological personality traits from the *Diagnostic and Statistical Manual of Mental Disorders, 5th edition* (DSM-5) alternative model of personality disorders. The scale is an abbreviated form of the initial 220-item extended form (Krueger et al., 2012). In this study, the Italian short form was used (PID-5; Fossati et al., 2024). The scale consists of 25 items with a 4-point Likert response scale (0 = very false or often false; 3 = very true or often true), evaluating the five pathological personality traits: negative affectivity (“I worry about almost everything”), detachment (“I often feel like nothing I do really matters”), antagonism (“It’s no big deal if I hurt other people’s feelings”), disinhibition (“People would describe me as reckless”), and psychoticism (“I have seen things that weren’t really there”). Cronbach’s alpha coefficient was 0.79 for negative affectivity, 0.66 for detachment, 0.72 for antagonism, 0.78 for disinhibition, and 0.81 for psychoticism (Gomez et al., 2020). The total score can range from 0 to 75; the higher the scores, the greater the overall personality dysfunction. Each trait domain has a score ranging from 0 to 15; the higher the scores, the greater the dysfunction in that specific personality trait domain.

## 3. Results

First, we calculated the descriptive statistics. We provided measures of central tendency and variability for the variables collected, as well as data on the skewness and kurtosis of their distributions in order to check the adherence to the statistical assumptions of the tests we intended to use (Table 1).

As indicated in Table 1, the variables showed a normal distribution, except for BLS-23 and PID-5 Antagonism, which were log-transformed. We applied this adjustment to satisfy the assumptions of parametric analyses, so we proceeded with correlation analyses among the variables collected. Due to the log transformation of these variables, the interpretation of the coefficients changes slightly. Rather than reflecting absolute changes, the results now reflect percentage changes in the associated variables. Therefore, the results should be interpreted in terms of the percentage increase or decrease in the associated variables, which provides a better understanding of the proportional differences between the factors under study.

We proceeded to explore the degree of association between the variables using the Pearson coefficient. Table 2 shows the correlations between the different measures used and the various dimensions of personality disorder traits.

### 3.1. Correlations with DLB

Almost all correlations between Digital Life Balance and personality traits were negative and significant, ranging from −0.10 (NPI-13, Entitlement/Exploitativeness) to −0.24 (BSL-23 and PID-5 total). These findings suggest that psychopathological personality traits are, on average, associated with an imbalanced integration of online and offline lives. The exceptions to these observations are the Leadership/Authority and Grandiose/Exhibitionism dimensions of the NPI-13, which were found to be positively correlated but without statistical significance, and antagonism in the PID-5, for which the negative correlation is not significant. Overall, these results suggest that higher levels of borderline symptoms (i.e., BLS-23) and traits of negative affectivity, detachment, disinhibition, and psychoticism as indicated by PID-5 and its total scores, as well as higher levels of narcissistic traits such as the Entitlement/Exploitativeness factor of the NPI-13, correspond on average to lower levels of DLB.

### 3.2. Correlations with IAS, BSMAS, SABAS, and GAS

Our sample showed specific differences in the relationships between the variables, but a trend emerged. Indeed, consistent positive and significant correlations were found between IAS, BSMAS, and SABAS for the Entitlement/Exploitativeness dimension and the total of the NPI-13. These findings suggest that people with higher levels of narcissistic traits, especially manipulative ones, tend to report higher levels of Internet, social media, smartphone, and application addiction. By contrast, their correlations with the Leadership/Authority and Grandiose/Exhibitionism dimensions do not reach statistical significance, except for SABAS, which shows significant positive correlations with the Leadership/Authority factor, albeit to a weak extent. This result indicates that, on average, authoritative and exhibitionistic traits are not associated with patterns of technological addiction. Nevertheless, these traits may be related to an increased risk of smartphone addiction. The correlations between IAS, BSMAS, SABAS, and NPI-13 range from 0.09 (correlation between IAS and NPI-13 total) to 0.21 (correlation between SABAS and Entitlement/Exploitativeness). This shows a weak to medium level of association, which warrants caution when interpreting the results. Conversely, the GAS does not show any correlation with the NPI-13.

Moreover, all the IAS, BSMAS, SABAS, and GAS scales show a positive and significant correlation with the BLS-23, ranging from 0.24 (GAS) to 0.35 (SABAS), indicating the medium entity of associations.

Similarly, in our sample all the IAS, BSMAS, SABAS, and GAS scales correlate positively and significantly with the negative affectivity, detachment, disinhibition, psychoticism, and total scores of the PID-5. Exceptions are observed for the antagonism dimensions, where positive correlations are significant only for IAS and SABAS. The correlations between these variables range from 0.11 (correlation between antagonism and SABAS) to 0.36 (correlation between negative affectivity and BSMAS), thus from a weak to moderate extent.

Overall, these findings indicate that higher levels of borderline symptoms (i.e., BLS-23), general narcissistic traits as indicated by the NPI-13 total score and those specifically described by its Entitlement/Exploitativeness dimension, as well as higher levels of almost all the personality traits assessed by the PID-5, on average correspond to higher levels of addictive patterns for the Internet (i.e., IAS), social networks (i.e., BSMAS), smartphones and applications (i.e., SABAS), and video games (i.e., GAS).

Since our analysis is based on correlations, we cannot determine if the examined personality traits directly lead to DLB or different forms of tech-related addiction. Consequently, no causal relationships can be established. Additionally, although online surveys help reach more people and encourage honesty (Donath, 1998), they may be affected by distractions, technical problems, or environmental interference, which can impact the validity of responses. Additionally, the low prevalence of gaming could be considered a limitation of the study. These aspects must be considered when interpreting the results.

### 3.3. Linear Discriminant Analysis (LDS) and Network Analysis

Linear Discriminant Analysis (LDA) was conducted to differentiate Digital Life Balance (DLB) status based on psychopathological personality traits. Specifically, the DLB variable was categorized into two groups: values below the first quartile, representing low Digital Life Balance, and values above the third quartile, indicating high Digital Life Balance. An LDA analysis was performed to determine if specific psychopathological personality traits could distinguish between people with low and high Digital Life Balance (DLB) levels. The analysis focused on the extremes of the DLB quartile distribution to highlight contrasts between groups with low and optimal digital life management and to detect the psychopathological traits that best distinguish these two profiles. However, an LDA analysis only reveals linear relationships and focuses on group differences. The association between DLB and psychopathological traits may be more complex, involving indirect or nonlinear associations. For this reason, a Network Analysis (NA) was performed to investigate the relationship between DLB and psychopathological personality traits more deeply. Given the potential nonlinearity of these relationships, NA was used to determine if the previous analysis revealed DLB-related outcomes that linear models and traditional analyses might miss. Thus, it can provide a deeper understanding of how digital life balance issues are part of and an expression of broader personality functioning. On LDA analysis, the model showed moderate discriminatory ability with an Area Under the Curve of 66.9% and an accuracy of 61.3%. The model performed better in identifying individuals with low levels of DLB than those with higher levels. Specifically, the precision estimates were 77.3% for individuals with high DLB, 52.5% for those with low DLB, and 66.9% for the total group. As shown in Table 3, of the personality traits, PID-5 disinhibition had the strongest influence on DLB, with a linear discriminant coefficient of −0.555.

The results of the LDA indicate a clear distinction between individuals with higher and lower Digital Life Balance. However, to explore the variables from a continuous perspective and account for potential nonlinear relationships, Network Analysis (NA) was conducted.

**Figure 1:**
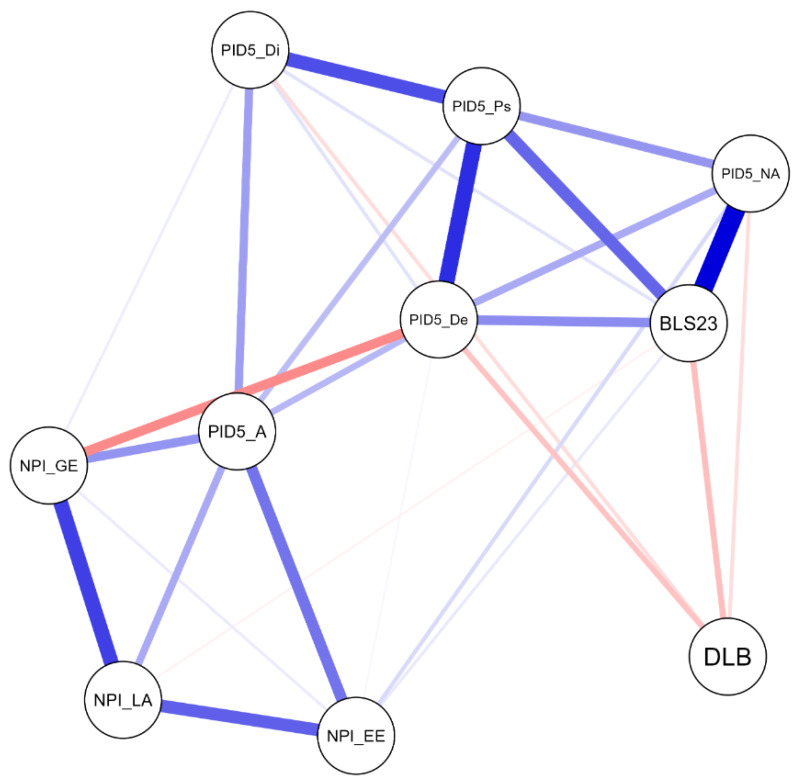
Network plot. Note: NPI_LA = Leadership/Authority; NPI_GE = Grandiose/Exhibitionism; NPI_EE = Entitlement/Exploitativeness; BLS = Borderline Symptom List (log transformed); PID5_NA = negative affectivity; PID5_De = detachment; PID5_A = antagonism; PID5_Di = disinhibition; PID5_Ps = psychoticism; Red lines = negative associations; Blue lines = positive associations.

**Figure 2:**
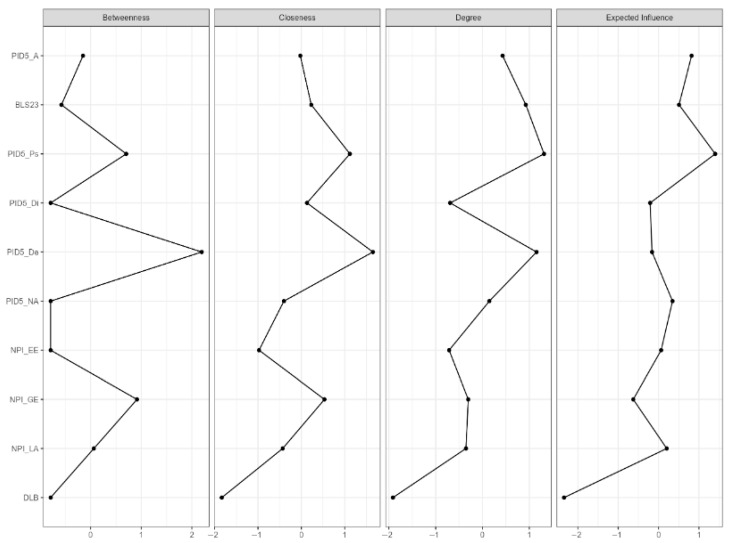
Centrality plot. Note: NPI_LA = Leadership/Authority; NPI_GE = Grandiose/Exhibitionism; NPI_EE = Entitlement/Exploitativeness; BLS = Borderline Symptom List (log transformed); PID5_NA = negative affectivity; PID5_De = detachment; PID5_A = antagonism; PID5_Di = disinhibition; PID5_Ps = psychoticism.

The Network Analysis (NA) of the entire sample revealed the presence of 10 nodes with a sparsity value of 0.33 (Figure 1). The NA shows how the different personality traits are associated with each other and with DLB. Overall, it highlights complex interrelationships among personality traits, with PID-5 Detachment emerging as the most central node in the network (Figure 2), and DLB also playing a significant role. As shown in Table 4, PID-5 Detachment exhibits negative associations with DLB (r = −0.082, *p* < 0.05), suggesting that people who tend to withdraw from social and emotional experiences may struggle to maintain a harmonious balance between their online and offline lives. Moreover, PID-5 Detachment exhibits negative associations also with NPI-Grandiose/Exhibitionism (r = −0.158, *p* < 0.05), indicating that people with high detachment tendencies may be less inclined to seek attention or engage in exhibitionistic behaviors. Conversely, PID-5 Detachment shows positive associations with PID-5 Psychoticism (r = 0.278, *p* < 0.05), BLS-23 (r = 0.156, *p* < 0.05), and PID-5 Negative Affectivity (r = 0.118, *p* < 0.05). These associations imply that people with higher detachment levels may be more prone to experience unusual perceptions, thoughts, and behaviors (i.e., psychoticism) and greater emotional instability (i.e., negative affectivity). They may also exhibit impulsivity and interpersonal difficulties, consistent with borderline personality features (i.e., BLS-23).

Additionally, BLS-23 also showed a negative association with DLB (r = −0.089, *p* < 0.05), indicating that people with higher borderline traits may be more likely to experience an imbalance in managing their digital and offline lives.

Overall, these findings suggest that DLB should be considered part of a broader network of personality traits and psychological functioning rather than an isolated behavior. Specifically, when this network includes psychopathological traits, they may negatively impact an individual’s ability to maintain harmony between digital and real-life domains.

## 4. Discussion

The Internet has increasingly become an integral part of individuals’ daily lives (Duradoni et al., 2024a; Kaess et al., 2014; Lopez-Fernandez, 2015; M. W. Zhang et al., 2017), and it is now considered an indispensable asset for living in the contemporary world (Kaess et al., 2014; Lopez-Fernandez, 2015). Its widespread use has brought various benefits, such as improved access to online information and facilitated social communication (M. W. Zhang et al., 2017), making services increasingly available and closer to people (e.g., learning, telemedicine, home banking) (Duradoni et al., 2024a). However, this complex framework of social and personal life changes also puts people at risk for dysfunctional use of the Internet and new technologies, potentially impacting their balance between online and offline life, as subsumed under the concept of Digital Life Balance (Duradoni et al., 2022). Psychological research in the area of Internet addiction, for example, has attempted to unravel these dynamics, and extensive evidence has been gathered on the relationship with mood disorders (Xie et al., 2023; M. Zhang & Bian, 2021), where depression has emerged as a potential predictor of mobile and social media addiction in different countries (Dailey et al., 2020; Haand & Shuwang, 2020; Jamir et al., 2019), and anxiety patterns may also contribute (Şentürk et al., 2021). People with these characteristics seem to find in new technologies an opportunity to escape from negative moods and to interact with others in a different way than from face-to-face communication (Aydin et al., 2020). Instead, there is still a paucity of literature on the relationship between Internet addiction and personality disorders. Therefore, the present study aimed to fill this gap by examining the association between different types of addiction (i.e., Internet, social media, smartphones and applications, and gaming) and narcissistic personality traits, borderline personality traits, and the PID-5 personality traits.

Overall, our study showed that on average, high scores on maladaptive personality traits correlated with lower DLB scores and higher levels of addictive patterns for the Internet (i.e., IAS), social networks (i.e., BSMAS), smartphones and applications (i.e., SABAS), and video games (i.e., GAS).

As we assumed in our first hypothesis (H1), the results suggested that higher levels of borderline personality traits correspond, on average, to lower levels of DLB and higher levels of Internet addiction scores. Moreover, positive and significant correlations were seen between borderline personality traits and BSMAS, SABAS, and GAS, suggesting their association with more dysfunctional patterns of technology use. This finding is consistent with previous research, which underlines that borderline traits are often associated with risk and actual dysfunctional Internet and technology uses by providing regulation of negative moods and emotions (Caplan, 2002; Dalbudak et al., 2014; W.-H. Lu et al., 2017), psychological distress (Wu et al., 2016), and compensation for social skills (Wu et al., 2016). In addition, this study partially supports our second hypothesis (H2) in that people with higher narcissistic scores on the NPI-13 reported lower DLB scores, but this correlation is significant only for the Entitlement/Exploitativeness factor. Then, as expected, higher levels of narcissistic personality traits tended to correlate with higher scores of Internet addiction as assessed by the IAS, although this positive correlation is significant only for the total score and the specific Entitlement/Exploitativeness factor. Similarly, other studies have shown the relevance of this specific factor, finding it to be predictive of the desire for likes online and linked to angry and vengeful behaviors when people do not respond to their online prompts (Zell & Moeller, 2017). Regarding DLB, only the exploitative dimension of the NPI-13 showed a significant, albeit weak, negative correlation with DLB. The total score and other dimensions (e.g., leadership/authority, grandiosity/exhibitionism) did not. These results suggest that the more manipulative and maladaptive facets of narcissism may impact digital life balance more than narcissism as a whole. In contrast, traits such as grandiosity and authority may involve more adaptive features, such as self-esteem and ambition, which could support more goal-directed and instrumental technology use. Network analysis supports this perspective, as NPI-13 traits were not directly linked to DLB but rather to other traits, such as antagonism and detachment. Furthermore, given the weak association that emerged, variables such as perceived social support, online social capital, platform type, and the size of one’s digital community may mediate this relationship. For example, people high in grandiosity may not exhibit imbalanced digital habits if they lack a strong online social capital or a supportive network, instead expressing their traits in offline contexts. Future research is needed to explore these potential mediation effects.

The analysis of the subdimensions of the NPI-13 and the other types of addiction showed a more differentiated picture. In fact, the correlations, although all positive, were significant only for the NPI-13 total score and the Entitlement/Exploitativeness factor with the BSMAS and SABAS, and also for the Leadership/Authority factor with the SABAS. Conversely, none of the specific narcissistic traits examined significantly correlated with the GAS. The absence of correlation between NPI-13 factors and the GAS contrasts with previous findings, for which the need for wealth and power would drive and reinforce the gaming behavior of people with narcissistic traits, also enhancing their self-esteem and social status (Kim et al., 2008). One possible explanation may lie in the characteristics of the sample. While 34.6% reported playing at least one video game, this does not imply regular or problematic use. This could limit the ability to detect associations with narcissistic traits, while it was sufficient to reveal stronger links with more pronounced traits, such as borderline tendencies, detachment, and disinhibition. Furthermore, the relationship between narcissism and gaming may depend on unmeasured mediating factors (e.g., gaming motivations, game types, or perceived online status and support), as suggested by some studies (Kircaburun et al., 2018).

Furthermore, in our sample, higher levels of disinhibition (H3), negative affectivity (H4), detachment (H5), psychoticism (H7), and PID-5 total scores corresponded on average to lower levels of DLB and higher levels of Internet addiction, thus confirming all the related hypotheses. Nevertheless, the results on detachment (H5) should be interpreted with caution, as this dimension exhibits lower reliability compared to the other Pid-5-BF factors. Exceptions are observed for the antagonism dimension, which did not show a significant correlation with DLB, preventing confirmation of part of the sixth hypothesis (H6). This result may be interpreted in light of the fact that the personality trait of antagonism is characterized, among other features, by a tendency toward deceitfulness and grandiosity (Krueger et al., 2012). Consequently, people with antagonistic personality traits may be less inclined to truthfully report their perception of the balance between online and offline life. This could have potentially altered the findings.

Overall, the presence of weak correlations (e.g., between narcissism and DLB, as well as between addictive measures and antagonism and disinhibition) warrants caution when interpreting the findings. This suggests to clinical interventions that, although these associations may not represent solid, established behaviors, they should be carefully considered when assessing general personality functioning.

To further deepen these findings, we conducted linear discriminant analysis (LDA) to assess the linear continuous relationships within the data and network analysis (NA) to identify potential non-linear and continuous patterns.

With regard to the former, the results confirmed and extended the correlational findings, highlighting traits such as disinhibition in particular, but also grandiosity, exploitation, and detachment as having a significant impact on lower levels of DLB. However, the LDA suggested the possible existence of a non-linear relationship between the variables. In this line, the NA revealed complex, predominantly positive, non-linear relationships between the traits. Notably, DLB emerged as an important node within the network, being largely negatively influenced by personality traits, thus further supporting the hypotheses of the studies. In particular, PID-5 detachment emerged as the most central factor in the network, showing a positive association with BLS-23 and a negative association with DLB, reinforcing its pivotal role in the observed personality network.

Some trends emerged in the correlations between the personality traits considered and different addictions. Specifically, BLS-23 and PID-5 dimensions positively correlated with social media, smartphones and applications, and gaming addictions. Again, the only exception is for the antagonistic trait, as the correlations with BSMAS and GAS scores are not supported by statistical significance. These associations may find explanation in the framework of the theory on compensatory Internet use (Kardefelt-Winther, 2014), glimpsing that features of online platforms provide a fertile background to help manage specific challenges of people presenting these traits. With respect to disinhibition, the present results are consistent with the hypothesis that the immediacy of online interactions would facilitate impulsive agency, thereby reducing the state of tension as a coping mechanism for the impulsive need to act (Gervasi et al., 2017). Similarly, the correlation with the negative affectivity component may relate, as already suggested (Leigh & Clark, 2018), to the absence of direct feedback that would allow a better venting of negative affective states. At the same time, the opportunity to handle limited interactions in a controlled environment could contribute to managing emotional activation issues (Müller et al., 2021), thereby facilitating the expression of the detachment trait, as observed in this study. Finally, as initially supposed, the Internet and social media would be a prolific ground to find confirmation (e.g., thanks to the filter bubble effect) and communities of like-minded people (e.g., through the echo chamber effect), providing together support to the need for social identity in people with psychotic traits, who, by contrast, often face social disapproval and frustration in offline life (Reinhard et al., 2022). In summary, these findings underscore the importance of considering individual personality differences when analyzing ICT usage patterns. Dysfunctional relationships with digital tools can provide valuable insight into personality traits, suggesting how these traits manifest and are reinforced in these relational dynamics with technology. The results encourage viewing unbalanced digital habits as expressions of deeper psychological needs that may undermine well-being, in addition to behaviors that require intervention. Specifically, certain psychopathological traits may influence how people use technology as a means of regulation, compensation, or self-expression. At the same time, these insights underline the importance of personalized approaches to assessment, prevention, and intervention strategies aimed at fostering healthier digital habits and improving overall psychological health.

### 4.1. Implications of the Results

The findings of this study reflect both theoretical and practical implications. From a theoretical perspective, they contribute to framing the relationship between disordered personality traits and addictions to new technologies, confirming the role of a potential vulnerability given by certain configurations of traits, but also adding to the literature the function of the dynamics of need compensation and satisfaction. That is, the results help to clarify that patterns of addiction may not only be promoted by a particular set of psychopathological traits that may predispose people to interact with and within new technologies in a certain way. Rather, the findings theoretically suggest that addictions in these domains may also be facilitated by specific drives to satisfy different human needs, for which they provide opportunities for fulfillment. These considerations thus add specific insights on personality traits to the Psychology of Harmony and Harmonization, further supporting the role of highly specific needs that may be frustrated in real-life settings, thus encouraging people with certain traits to compensate for their satisfaction online, where they may find fertile conditions for doing so. In particular, the factors that show the strongest correlations in this study are composed of subdimensions that relate to two major macro-categories of needs, namely sociality and control needs.

Elements of control are reflected in the observed importance of the Entitlement/Exploitativeness dimension of the NPI-13, which describes self-absorption, an inflated sense of one’s own abilities, and a belief that one deserves respect while being willing to manipulate others (Ackerman et al., 2011). People high in these traits are likely to engage in exploitative interpersonal behaviors (Brunell et al., 2013) and may seek to appear popular on social media, retaliating against those who do not respond to their desired attention (Zell & Moeller, 2017). Although these considerations are not further supported in this study by significant correlations with the factor of the Grandiose/Exhibitionism dimension of the NPI-13, the tendencies highlighted by the results for the Entitlement/Exploitativeness dimension may reflect a need to gain or maintain a sense of mastery and control over the social environment. At the same time, these dynamics may also be read as attempts to satisfy a search for specific patterns of social interaction (e.g., exploitative relationships) that may be less likely to be satisfied in real-life social situations.

In the direction of social needs, the BLS-23 also includes references to interpersonal problems and loneliness, which also suggests the potential influence of other motives that may drive the search for social contacts, namely seeking online social support to maintain self-esteem and self-image, to fill feelings of emptiness (T.-H. Chen et al., 2019), and to compensate for social skills (Wu et al., 2016), as supported by the existing literature. The valuable correlations we found with DLB in this sense further support this theoretical implication. Furthermore, returning to the need for control, it also seems possible to interpret it as a motivation to maintain mastery over one’s mental state by attempting to control impulsivity and emotional instability, as assessed by the BLS-23, by either venting or regulating them online.

In terms of practical implications, given that DLB may present differently depending on the personality traits exhibited by a person, these findings suggest the importance of individualized assessments and interventions that address the relationship between disordered personality traits and addictive patterns. In this sense, the results of this study could be informative for mental health professionals to develop targeted strategies that focus on needs, especially social and control needs, aiming to support their recognition and fulfillment through positive social ties, more functional coping strategies, and work on one’s own identity, at the same time balancing an excessive reliance on social media and digital platforms that may impair the online–offline life harmony.

Therefore, these findings could contribute to the formulation of guidelines for both preventive and therapeutic interventions, helping to identify factors and dynamics associated with risky technology use and monitor digital well-being in populations of people with disordered personality traits. These insights could benefit established psychotherapeutic approaches for personality disorders (e.g., dialectical behavior therapy, manual-assisted cognitive therapy, mentalization-based treatment, schema therapy, acceptance and commitment therapy, and systemic strategies), as well as interventions like systems training for emotional predictability and problem solving and social skills training (Crotty et al., 2024; Fayaz & Dhankar, 2025; Setkowski et al., 2023; Stoffers-Winterling et al., 2022). This is particularly pertinent for digital therapy formats, such as mobile apps, telehealth, Internet-based programs, and virtual reality (Drews-Windeck et al., 2023; Lindsay et al., 2024; Hudon et al., 2022). As these methods have demonstrated efficacy, it is crucial to address people’s dysfunctional relationships with technology. From this perspective, technology can be both a tool and a target of intervention. If clinicians understand the psychological processes underlying patients’ use of digital tools, they can effectively tailor interventions to address personality-related difficulties that manifest through problematic technology use.

Finally, the insights of this study could also inspire educational programs for awareness-raising projects aimed at adolescents and adults. For example, preventive programs could point to increasing awareness of content and dynamics shared on social media platforms (e.g., TikTok, Instagram) that may represent a risk factor for developing dysfunctional technology use by triggering individual personality traits and susceptibility. In this sense, awareness of mechanisms such as the filter bubble (Pariser, 2011) and the echo chamber effects (Sunstein, 2001), which expose people to like-minded communities, could help people recognize that their thoughts and opinions are not as widely shared as they appear. In fact, these dynamics can create a false sense of consensus. Additionally, increasing awareness that anonymity does not eliminate personal responsibility may mitigate disinhibited behaviors. Similarly, recognizing that frequent exposure to explicit violent content can encourage further disinhibition and the expression of negative emotions may lead to more responsible online behavior.

### 4.2. Limitations and Future Perspectives

Our study is not without limitations. First, it is important to recognize that while online surveys make it easier to collect data by reaching more people and encouraging honest responses (Donath, 1998), they might also be affected by bias like distractions, Internet issues, or environmental disturbances that can impact the validity of the questionnaire completion. Moreover, relying on self-report measures may have introduced bias, as participants may have provided socially desirable responses or lacked the self-awareness and introspective ability necessary for accurate reporting. Additionally, the self-report nature of the instruments used in this study may lead to an overestimation of symptoms. People with borderline traits often experience intense emotions, impulsivity, black-and-white thinking, an unstable sense of self, and a need for external validation. These characteristics could contribute to biased responses in self-report measures and should therefore be considered when interpreting them. Similarly, people with narcissistic traits may exhibit vulnerability, a grandiose self-perception, and a need for attention, all of which could bias their self-reported data.

Moreover, the voluntary nature of participation could have introduced self-selection bias, which should be considered. People with certain characteristics (e.g., a greater interest in the topic under study) may be more likely than others to participate in the study, which could affect the generalizability of the findings.

Additionally, the generalizability of our results may be limited, since the sample is mainly young (average age of 28.33 years), educated (52.60% have a secondary school diploma and 30.40% hold a bachelor’s degree), and largely made up of cisgender females (75.70%). Moreover, the generalizability of our results may be limited because the study did not analyze the impact of cultural factors. Ethnopsychiatry, indeed, explains how cultural factors influence the perception and classification of psychological distress (Gadit, 2003). At the same time, the same cultural factors also play a role in shaping relationships between individuals and society and consequently influence how ICTs are used (Erumban & De Jong, 2006). Future analyses should consider these aspects to improve the generalization of findings and deepen understanding of the role of cultural differences in dysfunctional technology usage.

Additionally, the potential influence of confounding variables, such as socio-economic status and pre-existing psychological conditions (e.g., depressive and anxiety patterns, which may frequently present together with personality disorders (Altaweel et al., 2023; Shah et al., 2023)), may represent a limitation that needs to be addressed in future research.

Another limitation of the study is the small number of participants who engage in gaming (approximately 34.6% of the sample). This warrants interpreting the related findings with caution.

Furthermore, since our analysis is based on correlations, we cannot conclusively determine whether the studied personality traits actually cause DLB or various types of addiction to new technologies and platforms. Therefore, it is impossible to determine a cause-and-effect relationship. Another limitation may be the distinctiveness of the constructs of the SABAS and IAS scales. Though they are designed to assess smartphone and Internet addiction separately, some items may overlap and address similar aspects of problematic technology use, leading to content redundancy. This may have affected the correlations with psychopathological traits by limiting the ability to distinguish the specific contribution of each scale. It may also point to a common core of technological addiction, which could have boosted the results. Although the observed correlations are weak to moderate, this limitation should be considered and overcome in future studies.

Future research is needed to refine these aspects. For example, future studies should consider more heterogeneous samples to improve the external validity of the results and take additional measures to minimize the potential impact of response bias on the outcomes. Moreover, longitudinal studies are needed to deepen the analysis by establishing causal relationships between the variables considered. They could also delve more deeply into a bio-psycho-social perspective of study, unraveling the influencing or mediating role of specific cultural, environmental, and social dynamics on this pattern of associations. Additionally, examining the social factors that contribute to Digital Life Balance could provide valuable insights. For example, exploring how individuals with strong offline social networks and supportive relationships are better equipped to meet their social needs both online and offline (Elgar et al., 2011) could offer a deeper understanding of the dynamics involved. Moreover, future research could examine the link between psychoticism traits and dysfunctional technology use by exploring the mediating role of mechanisms such as the filter bubble (Pariser, 2011) and echo chamber (Sunstein, 2001) effects. Researchers could test the hypothesis that these dynamics may reinforce maladaptive beliefs and foster a sense of belonging, thereby increasing online engagement and potentially reducing DLB. Longitudinal studies could also assess whether repeated exposure to confirmatory content and ideologically aligned communities can sustain or exacerbate unusual thoughts and behaviors, thereby contributing further to the online–offline imbalance. Additionally, future studies could examine whether these online dynamics could compensate for unmet social identity needs in offline contexts over time. This would provide a deeper understanding of the psychological processes that drive technology use among individuals with high psychoticism scores.

In addition, our study uses BSMAS to measure general social media addiction. Since each social media platform has unique features (e.g., TikTok, Instagram, X, Facebook, Snapchat, Reddit, and YouTube), future research could examine how psychopathological personality traits and DLB may manifest and differ across platforms. From an individual–environment interactionist perspective (Lewin, 1943), dysfunctional usage patterns may vary by platform, with different digital spaces fulfilling different psychological needs. Investigating these dynamics could shed light on how DLB is shaped by personality functioning and specific digital environments.

Additionally, qualitative studies could also extend the comprehension of specific processes and needs that drive people’s online behaviors.

For future lines of research, then, studies should explore factors such as narcissistic vulnerability (Ksinan et al., 2021; Wink & Donahue, 1997; Zerach, 2021) and the degree of identity impairment (T.-H. Chen et al., 2019), as these factors may contribute to the risk of developing dysfunctional or addicted Internet use.

## 5. Conclusions

Nowadays, with the widespread use of the Internet and new technologies, the risk of dysfunctional patterns of use, such as addiction, has increased. The aim of this study was to evaluate the relationships between Internet addiction and psychopathological personality traits (i.e., cluster C in particular).

Our findings showed a positive correlation between higher levels of addictive patterns of Internet use (i.e., IAS), social networks (i.e., BSMAS), smartphones and applications (i.e., SABAS), and video games (i.e., GAS) and higher levels of borderline symptoms. Moreover, the results showed that general narcissistic traits and those specifically described by the Entitlement/Exploitativeness dimension, as well as higher levels of personality traits assessed by the PID-5, on average correspond to higher levels of addiction to the Internet (i.e., IAS), social networks (i.e., BSMAS), smartphones and applications (i.e., SABAS), and video games (i.e., GAS).

These findings need to be further investigated, as they may contribute to the formulation of guidelines for preventive interventions that help identify factors associated with dysfunctional use of new technologies in people with personality disorders. Overall, the findings suggest to clinics that dysfunctional relationships with digital technologies and difficulty maintaining a balance between online and offline life seem to be deeply embedded in broader personality functioning. On the one hand, this invites assessment of underlying personality traits in cases of significant digital dysfunction, promoting targeted screening. Conversely, when a personality disorder has already been recognized, it is important to observe how such a configuration may manifest in people’s interactions with digital tools. In this sense, these findings may also contribute to the implementation of tailored therapeutic interventions based on the satisfaction of frustrated needs, particularly social and control needs, through functional coping strategies and positive social ties, rather than through excessive use of social media and new technologies.

## Figures and Tables

**Table 1:** Descriptive statistics of the study variables.

Variable	Min–Max	Mean (s.d.)	Skew.	Kurt.
NPI-13 (Leadership/Authority)	0–4	0.91 (1.16)	1.16	0.36
NPI-13 (Grandiose/Exhibitionism)	0–5	1.32 (1.39)	0.84	−0.23
NPI-13 (Entitlement/Exploitativeness)	0–4	1.10 (0.98)	0.62	−0.27
NPI-13 (Total)	0–12	3.33 (2.53)	0.87	0.36
BSL-23	0–3.84	0.78 (0.72)	1.60	2.71
PID-5 (Negative Affectivity)	0–15	7.00 (3.33)	−0.22	−0.71
PID-5 (Detachment)	0–13	3.86 (2.91)	0.64	−0.12
PID-5 (Antagonism)	0–14	2.48 (2.43)	1.21	1.68
PID-5 (Disinhibition)	0–13	3.45 (2.68)	0.71	−0.08
PID-5 (Psychoticism)	0–13	3.59 (3.08)	0.77	−0.01
PID-5 (Total)	0–59	20.38 (10.03)	0.31	−0.01
DLB	4–28	17.83 (5.16)	−0.18	−0.23
IAS	6–29	13.25 (4.18)	0.39	0.03
BSMAS ^✦^	6–30	12.68 (4.49)	0.65	0.19
SABAS	6–36	12.55 (5.30)	0.91	0.85
GAS ^⧫^	8–34	17.76 (5.83)	0.23	−0.72

Note: s.d.: standard deviation; Skew: skewness; Kurt.: kurtosis; N = 690; ^✦^: N = 655; ^⧫^: N = 235.

**Table 2:** Correlations among the study variables.

Variable	DLB	IAS	BSMAS ^✦^	SABAS	GAS ^⧫^
NPI-13 (Leadership/Authority)	0.03	0.04	0.05	0.08 *	0.12
NPI-13 (Grandiose/Exhibitionism)	0.04	0.01	0.04	0.04	0.03
NPI-13 (Entitlement/ Exploitativeness)	−010 *	0.16 ***	0.17 ***	0.21 ***	0.10
NPI-13 (Total)	−0.01	0.09 *	0.11 ***	0.14 ***	0.12
BSL-23 ^✤^	−0.24 ***	0.31 ***	0.34 ***	0.35 ***	0.24 ***
PID-5 (Negative Affectivity)	−0.21 ***	0.30 ***	0.36 ***	0.33 ***	0.14 *
PID-5 (Detachment)	−0.23 ***	0.27 ***	0.15 ***	0.25 ***	0.24 ***
PID-5 (Antagonism) ^✤^	−0.03	0.14 **	0.07	0.11 *	0.07
PID-5 (Disinhibition)	−0.16 ***	0.16 ***	0.14 ***	0.18 ***	0.28 ***
PID-5 (Psychoticism)	−0.15 ***	0.18 ***	0.20 ***	0.20 ***	0.17 ***
PID-5 (Total)	−0.24 ***	0.32 ***	0.29 ***	0.34 ***	0.28 ***

Note: N = 690; ^✦^: N = 655; ^⧫^: N = 235; ^✤^: the variable was log-transformed due to a non-normal distribution. *** = *p* < 0.001; ** = *p* < 0.01; * = *p* < 0.05.

**Table 3:** Standardized discriminant function coefficients.

Predictors	Coefficient
NPI-13 (Leadership/Authority)	0.336
NPI-13 (Grandiose/Exhibitionism)	−0.298
NPI-13 (Entitlement/Exploitativeness)	−0.391
BLS-23 ^✤^	−0.262
PID-5 (Negative Affectivity)	−0.148
PID-5 (Detachment)	−0.286
PID-5 (Antagonism) ^✤^	0.181
PID-5 (Disinhibition)	−0.555
PID-5 (Psychoticism)	−0.109

Note: ^✤^: the variable was log-transformed due to a non-normal distribution.

**Table 4:** Network weight matrix (n = 711).

Variable	1	2	3	4	5	6	7	8	9	10
1—DLB	0.000	0.000	0.000	0.000	−0.089	−0.047	−0.082	0.000	−0.047	0.000
2—NPI-13 (Leadership/Authority)	0.000	0.000	0.215	0.215	−0.016	0.000	0.000	0.117	0.000	0.000
3—NPI-13 (Grandiose/Exhibitionism)	0.000	0.255	0.000	0.028	0.000	0.000	−0.158	0.147	0.025	0.000
4—NPI-13 (Entitlement/Exploitativeness)	0.000	0.215	0.028	0.000	0.030	0.052	0.000	0.189	0.000	0.010
5—BLS-23 ^✤^	−0.089	−0.016	0.000	0.030	0.000	0.342	0.156	0.000	0.040	0.206
6—PID-5 (Negative Affectivity)	−0.047	0.000	0.000	0.052	0.342	0.000	0.118	0.001	0.008	0.143
7—PID-5 (Detachment)	−0.082	0.000	−0.158	0.000	0.156	0.118	0.000	0.099	0.040	0.278
8—PID-5 (Antagonism) ^✤^	0.000	0.117	0.147	0.189	0.000	0.001	0.099	0.000	0.131	0.089
9—PID-5 (Disinhibition)	−0.047	0.000	0.025	0.025	0.040	0.008	0.040	0.131	0.000	0.238
10—PID-5 (Psychoticism)	0.000	0.000	0.000	0.000	0.206	0.143	0.278	0.089	0.238	0.000

Note: ^✤^: the variable was log-transformed due to a non-normal distribution.

## Data Availability

The data presented in this study are available on request from the corresponding author.
